# The Role of Environmental Tax in Alleviating the Impact of Environmental Pollution on Residents’ Happiness in China

**DOI:** 10.3390/ijerph16224574

**Published:** 2019-11-19

**Authors:** Yu Liu, Rong-Lin Li, Yang Song, Zhi-Jiang Zhang

**Affiliations:** 1 College of Management, Wuhan Institute of Technology, Wuhan 430205, China; lyu429@163.com (Y.L.); ronglin722@163.com (R.-L.L.); summersong95@163.com (Y.S.); 2 Department of Preventive Medicine, School of Health Sciences, Wuhan University, Wuhan 430072, China

**Keywords:** air pollution, water pollution, happiness, environmental tax, moderating effect

## Abstract

*Background*: Environmental tax has been implemented by the government in response to the demands of the residents to control environmental pollution. However, a tax has a wide effect on many interacting aspects of the society. It remains unknown whether enacting an environmental tax for the government can improve the residents’ happiness. This study aimed to examine the impact of air and water pollution on residents’ happiness and evaluate whether an environmental tax can alleviate the impact of air and water pollution on residents’ happiness. *Methods*: Based on the 2015 Chinese General Social Survey Data, 28 provinces in China were divided into two categories according to their environmental tax rates: baseline-tax areas (*n* = 13) and high-tax areas (*n* = 15). The ordered probit model was used to analyze the impact of air and water pollution on the residents’ happiness in baseline-tax areas and high-tax areas, respectively. The Chow Test was used to test whether the impact of environmental pollution on happiness was different between baseline-tax areas and high-tax areas. *Results:* The impact of air pollution on residents’ happiness was statistically significant in the baseline-tax areas (coefficient −0.162, 95% confidence interval (CI) −0.239, −0.086, *p* < 0.001), but the significance was weakened in the high-tax areas (coefficient −0.030, 95% CI −0.060, 0.000, *p* = 0.051). The Chow Test showed that the absolute value of the regression coefficient in the baseline-tax areas was significantly higher than the value in the high-tax areas (F = 12.712, *p* < 0.001). Similarly, the impact of water pollution on residents’ happiness was statistically significant (coefficient −0.264, 95% CI −0.353, −0.174, *p* < 0.001) in the baseline-tax areas and in the high-tax areas (coefficient −0.063, 95% CI −0.091, −0.035, *p* < 0.001), but the Chow Test showed that the absolute value of the regression coefficient in the baseline-tax areas was significantly higher than the value in the high-tax areas (F = 13.758, *p* < 0.001). *Conclusions*: Both air and water pollution impair residents’ happiness. The present study shows for the first time that enacting an environmental tax significantly alleviates the negative effect of air and water pollution on residents’ happiness. The findings of the present study provide empirical evidence for the government to levy environmental tax.

## 1. Introduction

The rapid economic growth in China has been accompanied with environment degradation. Previous studies show that self-reported life satisfaction has not increased in China as much as would be expected [1]. Environmental pollution can obviously, at least partly, explain this paradox [2,3,4,5,6]. 

Air and water pollution, as the major cause of environmental deterioration, seriously affect residents’ psychological and physical health status [7]. It has been reported that air pollution is associated with increased annoyance [8], anxiety [9], and, more devastatingly, several mental disorders, such as depression [10,11] and schizophrenia [12]. Furthermore, air pollution causes a variety of diseases, e.g., pneumonia, asthma, stroke, and heart diseases [13]. Water pollution mainly includes industrial wastewater discharge, pesticide penetration and urban domestic sewage discharge. Water pollution produces bad smells, odors, and strange colors, which stimulate the senses of the residents, weaken the residents’ desire to travel, and impair the emotions of the residents [14]. In addition, water pollution increases the costs of water consumption and leads to inconvenience to residents’ lives [15]. Furthermore, toxic substances in contaminated water may be absorbed into the body through drinking or food consumption, and threaten the health of residents, e.g., liver cancer [16].

Given that health status and psychological well-being are two important factors affecting happiness, the impact of air and water pollution on residents’ life satisfaction have been a hot topic [6,11]. Most studies demonstrate that environment pollution has a negative impact on residents’ happiness [3,4,17,18]. Several studies explored the relationship between environmental pollution and happiness in China [1,19,20,21,22]. Through an analysis of 210 million geotagged Weibo tweets in China, Zheng et al. revealed that air pollution was associated with lower happiness expressed in tweets [22]. Zheng used data on Chinese industrial air waste and water waste to explore the relationship between pollution and the happiness of residents in China, and it was found that there is a significant negative correlation between them [14].

It has been proposed that the government can address the externality problem of pollution by implementing an appropriate tax in order to internalize the social cost of polluting emissions [23,24]. Environmental taxes and fees have been used worldwide in response to the deteriorating quality of the environment. Since then, there have been a lot of empirical studies on the effect of environmental tax on pollution [25,26,27,28,29]. Gaube found that environmental taxes were useful in improving environmental quality on the condition of the positive marginal benefits of environmental taxes [30]. However, some studies obtained an opposite conclusion. For example, Metcalf used the input–output framework with fifty-eight industry sectors to estimate the impact of environmental tax, and found that the impacts of such a reform were likely to be modest [31]. Gemechu et al. found that both the environmental target and economic target were very hard to achieve in Spain [32].

It has been 40 years since China launched an environment tax/fee system. It is possible to evaluate the effectiveness of the policy on environmental protection and associated social welfare in China. There are limited studies focused on this topic and they have reached different conclusions [33]. Some studies reported that the environmental tax system has exerted an effect on controlling air pollution [34,35,36]. Wang and Wheeler found that air and water pollution charges in China have exerted a positive effect on sewage control [37]. Li and Masui also found that the environmental tax could help reduce emissions of most kinds of pollutants base on the computable general equilibrium (CGE) model [38]. Ren also affirmed that the market-oriented environmental policy tools were beneficial to improving regional ecological efficiency [39]. Other studies reported that environmental tax failed to play a role in curbing industrial pollution [40,41]. According to Shibli and Markandya, the pollutant charge was relatively ineffective in China, because it was casually dependent on the local government [42]. The effects of the environmental standard charge on air pollution reduction in Shandong Province in China were not significant in the studies by Yuan et al. [43] and Hui et al. [33].

Happiness is an important indicator reflecting the quality of people’s livelihood [1]. Improving happiness has become one of the most important tasks for the government. Few studies have examined whether levying environmental tax policies has an effect on residents’ happiness. Accordingly, this study aims to (1) examine the impact of air and water pollution on residents’ happiness in China; and (2) evaluate whether an environmental tax can alleviate the impact of air and water pollution on residents’ happiness based on the moderating effect model. The present study explores, for the first time, the impact of an environmental tax on the social system from the perspectives of residents’ happiness. The findings of the present study will testify the moderating effect of environmental tax and provide evidence for the government to levy environmental tax. This happiness analysis approach can provide theoretical evidence for improvement in environmental tax for the government.

The rest of the paper is organized as follows. Section 2 reviews the policy background. Section 3 introduces the methods of the moderating model. Section 4 analyses the empirical results. Section 5 discusses the conclusion and the probable mechanism. Section 6 is on the limitations of the study.

## 2. Policy Background

During the period 1978–2017, China established and carried out a sewage charge system. The rate of charge changed several times. Provincial governments decide their pollutant charge rates independently in China, although the baseline criterion is designed at the national level [33]. Table 1 lists the sewage charge rates for sulfur dioxide (SO_2_) in the air and Chemical Oxygen Demand (COD, an indirect indicator for determining the organic content in water systems, reflecting the overall status of water pollution) in the water for 28 provinces in 2014. In 2014, 13 of 28 provinces implemented a baseline-tax rate required by the central government, i.e., 0.6 yuan per pollution equivalent for SO_2_ and 0.7 yuan per pollution equivalent for the COD. The other 15 provinces used a rate of 1.2 yuan per pollution equivalent for SO_2_ and 1.4 yuan per pollution equivalent for COD. Beijing used a rate of 9.5 yuan per pollution equivalent for SO_2_ and 10 yuan per pollution equivalent for COD (Table 1). These areas were defined as high-tax areas for the present study. On 1 January 2018, an environmental tax system was enacted to replace the former sewage charge system. The tax rates for each pollutant were roughly the same as the former sewage charge rates.

## 3. Methods

### 3.1. Data Resource

Data on residents’ happiness were obtained from the latest Chinese General Social Survey Data (CGSS) 2015. The total sample size of the CGSS is 10,968, comprised of participants from 28 provinces of the 31 mainland provinces in China exclusive of Hainan, Xinjiang and Tibet, and is regarded as a nationally representative survey. For the present study, a total of 7 variables were extracted, i.e., happiness, gender, age, religion, marriage, health and income. After removing those with missing values for any of the 7 variables, there were 10,752 (98.1%) participants.

Emissions data on industrial SO_2_ and industrial COD in 28 provinces were derived from the China Environmental Statistical Yearbook (2015). As the CGSS was carried out in 2014, the data corresponded to the pollution and sewage charge rates in 2014. The SO_2_ and COD environmental tax rates were acquired from the publicly available official documents or website of each provincial government.

### 3.2. Model Specifications

The empirical model used in the present study was as follows:

(1) $$Happiness_{n}=\alpha_{1}\ln PollutionA_{ni}+\beta_{1}Y_{n}+\varepsilon_{1}\;$$

(2) $$Happiness_{n}=\alpha_{2}\ln PollutionW_{ni}+\beta_{2}Y_{n}+\varepsilon_{2\;}$$

In Equations (1) and (2), α and β are the regression coefficients of the equations, ε is the error term of the regression model, $Happiness_{n}$ represents the happiness of the n residents, $PollutionA_{ni}$ represents the degree of air pollution in the *i* province of the n residents, $PollutionW_{ni}$ represents the degree of water pollution in the *i* province of the *n* residents, and $Y_{n}$ represents the collection of control variables of the *n* residents. Residents’ happiness is an ordered variable, ranking between 1 and 5, so we use the ordered probit model to estimate Equations (1) and (2) [4].

### 3.3. Dependent Variables

Residents’ happiness (Happiness). We selected the 36th question in CGSS 2015: “Generally speaking, do you think your life is happy?”. There are five choices, i.e., very unhappy, unhappy, it is hard to say happy or unhappy, happy and very happy. The five choices were assigned values from 1 to 5, correspondently.

### 3.4. Independent Variables

Air pollution (PollutionA). Industrial SO_2_ emission is the main cause of the increase in PM2.5, and is more irritating to humans. Therefore, we selected industrial SO_2_ emission as a proxy variable for air pollution level [44]. The logarithmic transformation was used for industrial SO_2_ emission.

Water pollution (PollutionW). COD is widely used in water quality monitoring in China. We selected industrial COD emission as a proxy variable for water pollution levels. The logarithmic transformation was used for industrial COD emission.

### 3.5. Moderator Variables

The SO_2_ tax rates in 2014 were used as a moderator for the association between air pollution and residents’ happiness, because the CGSS 2015 was carried out in 2014. Table 1 lists the SO_2_ sewage taxation/charge rates in 28 provinces in 2014. A total of 13 provinces (called baseline-tax area) used 0.6 yuan/pollution equivalent, and the other 15 provinces (called high-tax area) used higher rates >0.6 yuan/pollution equivalent. Therefore, the moderator variable (Tax1) in the present study was assigned a value of 0 when the tax rate was 0.6 yuan/pollution equivalent and 1 when the tax rate was >0.6 yuan/pollution equivalent.

The COD tax rates in 2014 were used as a moderator for the association between water pollution and residents’ happiness. Table 1 lists the COD sewage taxation/charge rates in 28 provinces in 2014. A total of 13 provinces (called baseline-tax area) used 0.7 yuan/pollution equivalent, and the other 15 provinces (called high-tax area) used higher rates >0.7 yuan/pollution equivalent. Therefore, the moderator variable (Tax2) in the present study was assigned a value of 0 when the tax rate was 0.7 yuan/pollution equivalent and 1 when the tax rate was >0.7 yuan/pollution equivalent.

### 3.6. Control Variables

We controlled a set of factors that could affect subjective happiness. Previous studies at the individual level show that age, gender, marriage, religion, health and family income status are closely correlated with residents’ happiness [1,4,14]. Therefore, we used gender, age, age^2^, religion, marriage, health and income as control variables in the present study.

### 3.7. Chow Test

To test the difference between the coefficients from the model in the baseline-tax area and high-tax area, we used the Chow Test, which was widely used to test whether structural changes exist [45].

## 4. Results

### 4.1. Descriptive Analyses

In the present study, the proportion of males was 46.8% (Table 2). Age ranged from 17 to 93 years, with an average age of 49.4 years. The proportion of married participants was 89.0%. The resident self-reported health status is rated at 5 and the average health status is 3.608. The resident self-reported family income status is rated at 5 and the average income status is 2.652. The average residents’ happiness was 3.867. The average industrial SO_2_ emissions were 602,950 tons, and the average industrial COD emissions were 110,300 tons.

### 4.2. Empirical Results

We used the STATA software to perform an ordered probit regression analysis of Equations (1) and (2). The regression result was shown in Table 3. The emission of industrial SO_2_ in air pollution had a negative impact on residents’ happiness. The regression coefficient was −0.059 (95%CI −0.086, −0.031) with *p* < 0.001. The emission of industrial COD in water pollution had a negative impact on residents’ happiness. The regression coefficient was −0.089 (95%CI −0.116, −0.063) with *p* < 0.001. The regression result indicated that the emission of industrial SO_2_ and COD significantly reduced residents’ happiness, which was consistent with previous studies. In order to enhance the robustness of the regression result, the models contained the individual control variables, and the statistical significance of control variables was high, indicating that the effect of the regression analysis was robust, and the results of the individual feature control variables were basically similar to the previous studies [2].

### 4.3. Moderating Effect Results

In Table 4, the coefficient of the SO_2_ sewage charge in baseline-tax areas was −0.162 (95% CI −0.239, −0.086) and statistically significant (*p* < 0.001), supporting a negative effect of air pollution on residents’ happiness. Conversely, the coefficient of the SO_2_ sewage charge in high-tax areas was −0.030 (95% CI −0.060, 0.000), indicating a weakened significance (*p* = 0.051). The Chow Test showed that the two coefficients in baseline- and high-tax areas were statistically different (F = 12.712, *p* < 0.001).

The coefficient of the COD sewage charge in baseline-tax areas was −0.264 (95% CI −0.353, −0.174) and statistically significant (*p* < 0.001, Table 4), supporting a negative effect of water pollution on residents’ happiness (Table 4). In contrast, the coefficient of the COD sewage charge in high-tax areas was −0.063 (95% CI −0.091, −0.035) and statistically significant (*p* < 0.001). The Chow Test showed that the two coefficients in baseline- and high-tax areas were statistically different (F = 13.758, *p* < 0.001).

## 5. Discussion

This study evaluated whether environmental tax moderates the relation between environmental pollution and residents’ happiness. Our analyses were stratified according to the tax rates. We found that industrial SO_2_ emissions impair residents’ happiness, and the magnitude of influence was much greater in baseline-tax areas than in high-tax areas, indicating an alleviating effect of air pollution tax on the relationship between air pollution and residents’ happiness. Second, we found that industrial COD emissions impair residents’ happiness, and the magnitude of influence was much greater in baseline-tax areas than in high-tax areas, indicating an alleviating effect of water pollution tax on the relationship between water pollution and residents’ happiness.

One of the findings of this study is that air and water pollution have a significant negative impact on residents’ happiness. The findings are consistent with previous studies [1,4,18]. The direction of effect is the same, although the magnitude is different. Zheng et al. reported that industrial wastewater in 2008 had a significant negative effect on residents’ happiness, and the coefficient was −0.123 [14]. In contrast, the coefficient in our study is −0.089 (baseline- and high-tax areas combined). The reason may be that water pollution has been alleviated in recent years. The negative effect in the baseline-tax areas (coefficient −0.264) is greater than in Zheng’s results, and the effect in the high-tax areas (coefficient −0.063) is smaller than in Zheng’s results. Luechinger used the German Socio-Economic Panel (GSOEP) containing information on individual life satisfaction to examine the impact of air pollution on life satisfaction [46]. The regression coefficient is −0.005 based on panel data for the period 1985–2003 consisting of 29,246 individuals. Di Tella and MacCulloch found that the happiness responses of approximately 350,000 people living in the Organization for Economic Cooperation and Development (OECD) between 1975 and 1997 are negatively correlated with the level of SO_2_ emissions [47]. Their coefficient is −0.003. In contrast, the coefficient is −0.059 in the present study, which is greater than the results from Luechinger [46], Di Tella and MacCulloch [47]. The comparison between ours and other studies show that air pollution has a greater influence on residents’ happiness in China than in those countries.

The most important finding of the present study is that environmental tax plays a moderator role on the relationship between environmental pollution and residents’ happiness. Specifically, air and water pollution had a greater negative impact on residents’ happiness in the baseline-tax areas than in the high-tax areas, indicating a moderating effect of environmental tax on the negative impact of air and water pollution on residents’ happiness. The underlying mechanisms remain unclear but could be twofold. On the one hand, the taxation of pollutants can stimulate energy conservation and emission reduction [34,35], which in turn leads to decreased negative impact on residents’ happiness. On the other hand, the revenue of environmental tax may improve social welfare if appropriately used [26].

## 6. Conclusions

Air and water pollution have already become a tough issue in China. Sewage control and improving the environment need public regulation measures. The present study analyzed the moderating effect of environmental tax on the relationship between environmental pollution and residents’ happiness. Environmental pollution has a negative effect on residents’ happiness, and the present study shows for the first time that enacting an environmental tax significantly alleviates the negative effect of air and water pollution on residents’ happiness. The findings of this study have important policy implications. First, although the effect of environmental tax is controversial, the findings of the present study provide evidence for the government to levy environmental tax. Second, the optimal environmental tax criterion is still unclear, nevertheless, the empirical results may provide references for the policy makers. In China, local governments could raise the environmental tax rate, especially for the base-line tax areas. Local governments should exert a reasonable environmental tax rate to alleviate the effect of pollution on residents’ happiness. In order to protect the local economy, local governments do not have the incentive to raise the environmental tax rate. On the one hand, the central government could raise the baseline-tax rate. On the other hand, residents’ happiness could be taken into account by local officials.

Future research could be undertaken to study the spatial interactions of tax rate changes between regions on pollution emission. Disentangling the potentially confounding effect of weather may be important for environmental pollution and happiness. Air pollution in Chinese cities is known to be highly sensitive to wind direction and speed, as pollutants are carried from neighboring cities. Some large watershed and lakes span several provinces, such as Yangtze River, Yellow River and Poyang Lake. Pollution upstream may affect the environment downstream. In this sense, there could be some potential spatial interactions. However, due to limited data, we did not consider spatial interactions in this paper.

## Figures and Tables

**Table 1:** The sewage charge rates for SO_2_ and Chemical Oxygen Demand (COD) in 28 provinces in 2014 in China (yuan/per pollution equivalent).

Province	SO_2_	COD	Province	SO_2_	COD
Tianjin	1.2	1.4	Henan	0.6	0.7
Shanghai	1.2	1.4	Hubei	0.6	0.7
Beijing	9.5	10	Hunan	0.6	0.7
Chongqing	0.6	0.7	Guangdong	1.2	1.4
Hebei	1.2	1.4	Sichuan	0.6	0.7
Shanxi	0.6	0.7	Guizhou	1.2	1.4
Liaoning	1.2	1.4	Jiangxi	0.6	0.7
Jilin	0.6	0.7	Yunnan	0.6	0.7
Heilongjiang	1.2	1.4	Shaanxi	0.6	0.7
Jiangsu	1.2	1.4	Gansu	0.6	0.7
Zhejiang	1.2	1.4	Qinghai	0.6	0.7
Anhui	1.2	1.4	Inner Mongolia	1.2	1.4
Fujian	0.6	0.7	Guangxi	1.2	1.4
Shandong	1.2	1.4	Ningxia	1.2	1.4

Note: data were obtained from the provinces’ environmental tax and sewage charge documents.

**Table 2:** Descriptive statistics.

	Variables	Measures	Mean	Standard Deviation	Minimum	Maximum
Dependent variable	Happiness	The five levels of respondents’ self-reported happiness	3.867	0.821	1	5
Independent variable	Air Pollution (PollutionA)	Industrial SO_2_ emissions	60.295	33.095	4.035	140
	Water Pollution (PollutionW)	Industrial COD emissions	11.030	5.694	0.605	23.550
Control variable	Gender	Male = 1; Female = 0	0.468	0.499	0	1
	Age	The age of respondents	49.402	16.895	17	93
	Age^2^	The square of age	2725.960	1708.599	289	8649
	Religion	Religion = 1; No religion = 0	0.109	0.312	0	1
	Marriage	Married = 1; Others = 0	0.890	0.313	0	1
	Health	The five levels of respondents’ health	3.608	1.075	1	5
	Income	The five levels of respondents’ family income status	2.652	0.717	1	5
Moderator variable	Tax1	Sewage charges (SO_2_)	1.338	1.894	0.6	9.5
	Tax2	Sewage charges (COD)	1.507	1.977	0.7	10

**Table 3:** Regression results of environmental pollution effect on residents’ happiness.

Dependent Variable		Happiness					
Independent Variable		PollutionA			PollutionW		
		Coefficient	95% CI	*p* Value	Coefficient	95% CI	*p* Value
		−0.059	(−0.086, −0.031)	0.000	−0.089	(−0.116, −0.063)	0.000
Control Variable	Gender	−0.077	(−0.120, −0.034)	0.000	−0.075	(−0.118, 0.032)	0.001
	Age	−0.030	(−0.038, −0.022)	0.000	−0.030	(−0.038, −0.022)	0.000
	Age^2^	0.000	(0.000, 0.001)	0.000	0.000	(0.000, 0.001)	0.000
	Religion	0.139	(0.071, 0.208)	0.000	0.154	(0.085, 0.223)	0.000
	Marriage	0.239	(0.152, 0.325)	0.000	0.249	(0.163, 0.336)	0.000
	Health	0.237	(0.215, 0.260)	0.000	0.237	(0.214, 0.259)	0.000
	Finance	0.416	(0.385, 0.447)	0.000	0.416	(0.384, 0.447)	0.000
Number of Observation		10752			10752		
Pseudo R^2^		0.065			0.066		
Log likelihood		−11,289.057			−11,275.926		
Prob > chi2		0.000			0.000		

**Table 4:** Environmental tax moderator effect test results.

Dependent Variable		Happiness											
Independent Variable/Group		PollutionA						PollutionW					
		Baseline-Tax Area			High-Tax Area			Baseline-Tax Area			High-Tax Area		
		Coefficient	95% CI	*p* Value	Coefficient	95% CI	*p* Value	Coefficient	95% CI	*p* Value	Coefficient	95% CI	*p* Value
		−0.162	(−0.239, −0.086)	0.000	−0.030	(−0.060, 0.000)	0.051	−0.264	(−0.353, −0.174)	0.000	−0.063	(−0.091, −0.035)	0.000
Control Variable	Gender	−0.106	(−0.169, −0.042)	0.001	−0.056	(−0.115, 0.002)	0.059	−0.105	(−0.169, 0.042)	0.001	−0.055	(−0.113, 0.004)	0.067
	Age	−0.030	(−0.042, −0.017)	0.000	−0.029	(−0.040, −0.018)	0.000	−0.029	(−0.042, −0.017)	0.000	−0.030	(−0.041, −0.019)	0.000
	Age^2^	0.000	(0.000, 0.001)	0.000	0.000	(0.000, 0.001)	0.000	0.000	(0.000, 0.001)	0.000	0.000	(0.000, 0.001)	0.000
	Religion	0.208	(0.104, 0.312)	0.000	0.069	(−0.023, 0.161)	0.140	0.229	(0.126, 0.333)	0.000	0.084	(−0.008, 0.176)	0.073
	Marriage	0.142	(0.003, 0.280)	0.046	0.295	(0.185, 0.405)	0.000	0.148	(0.009, 0.287)	0.037	0.311	(0.200, 0.421)	0.000
	Health	0.249	(0.217, 0.282)	0.000	0.225	(0.195, 0.256)	0.000	0.248	(0.216, 0.281)	0.000	0.225	(0.195, 0.256)	0.000
	Finance	0.461	(0.414, 0.509)	0.000	0.381	(0.340, 0.422)	0.000	0.463	(0.415, 0.511)	0.000	0.381	(0.340, 0.422)	0.000
Chow Test	F	12.712						13.758					
	*p* Value	0.000						0.000					
Obs		4973			5779			4973			5779		
Pseudo R^2^		0.0744			0.0579			0.0759			0.0592		
Log likelihood		−5142.1443			−6118.2294			−5134.2228			−6110.2975		
Prob>chi2		0.000			0.000			0.000			0.000		
